# Identification of TCP13 as an Upstream Regulator of *ATHB12* during Leaf Development

**DOI:** 10.3390/genes10090644

**Published:** 2019-08-26

**Authors:** Yoon-Sun Hur, Jiyoung Kim, Sunghan Kim, Ora Son, Woo-Young Kim, Gyung-Tae Kim, Masaru Ohme-Takagi, Choong-Ill Cheon

**Affiliations:** 1 Department of Biological Science and Research Institute of Women’s Health, Sookmyung Women’s University, Seoul 04310, Korea; 2 College of Pharmacy, Sookmyung Women’s University, Seoul 04310, Korea; 3 Bioproduction Department of Molecular Biotechnology, Dong-A University, Busan 49315, Korea; 4 Bioproduction Research Institute, National Institute of Advanced Industrial Science and Technology (AIST), Tsukuba, Ibaraki 305-8566, Japan; 5 Institute for Environmental Science and Technology (IEST), Saitama University, Saitama 338-8570, Japan

**Keywords:** leaf development, ATHB12, TCP13, cell expansion, upstream regulator

## Abstract

Leaves grow by distinct phases controlled by gene regulatory networks including many transcription factors. *Arabidopsis thaliana homeobox 12* (*ATHB12*) promotes leaf growth especially during the cell expansion phase. In this study, we identify TCP13, a member of the TCP transcription factor family, as an upstream inhibitor of *ATHB12*. Yeast one-hybrid screening using a 1.2-kb upstream region of *ATHB12* resulted in the isolation of TCP13 as well as other transcription factors. Transgenic plants constitutively expressing *TCP13* displays a significant reduction in leaf cell size especially during the cell expansion period, while repression of *TCP13* and its paralogs (*TCP5* and *TCP17*) result in enlarged leaf cells, indicating that TCP13 and its paralogs inhibit leaf development, mainly at the cell expansion phase. Its expression pattern during leaf expansion phase is opposite to *ATHB12* expression. Consistently, the expression of *ATHB12* and its downstream genes decreases when *TCP13* was overexpressed, and increases when the expression of *TCP13* and its paralogs is repressed. In chromatin immunoprecipitation assays using *TCP13-GFP* plants, a fragment of the *ATHB12* upstream region that contains the consensus sequence for TCP binding is strongly enriched. Taken together, these findings indicate that TCP13 and its paralogs inhibit leaf growth by repressing *ATHB12* expression.

## 1. Introduction

Leaves are plant organs essential for harvesting the light that provides energy for living organisms. The development of leaves involves complicated coordination of several factors including correct spatio-temporal transcriptional regulation of genes, hormonal control, and responses to environmental conditions [1,2,3,4,5]. After the formation of leaf primordia, which are groups of leaf founder cells on the flanks of shoot apical meristems, cell proliferation results in a relatively constant cell size. While cell division continues at the base of the leaf, cell expansion starts at the tip and moves to the base, forming the cell cycle arrest front [1,6,7]. At the same time, differentiation occurs to form specialized cells such as guard cells.

Homeobox genes affect plant development as well as the development of various animals [8,9]. Homeodomain-leucine zipper (HD-Zip) genes, a subset of homeobox genes with tightly linked leucine zipper motifs [10], are critical for plant development. They are classified into four distinct subfamilies (I–IV) of HD-Zips, many of which have been revealed to be critical for various aspects of plant development [11,12]. For example, some HD-Zip III proteins such as PHABULOSA/ATHB14, PHAVOLUTA/ATHB9 and REVOLUTA, regulate the formation of the adaxial domains of leaves [13,14]. *Arabidopsis thaliana* homeobox 2 (ATHB2) and ATHB4, HD-Zip II proteins, are induced by a low ratio of red to far-red light and contribute to the shade avoidance response, including hypocotyl elongation [15,16]. *ATHB2* expression is controlled by basic helix–loop–helix (bHLH) phytochrome-interacting factors PIF4 and PIF5, as Kunihiro et al. [17] showed that PIF5 binds to some of the G-box-rich regions of the *ATHB2* promoter. HD-Zip II proteins are also involved in the regulation of adaxial–abaxial patterning through repression of miR165/166 expression, together with HD-Zip III proteins [18]. *ATHB12*, an HD-Zip I gene, is primarily expressed in leaves and stems and is also inducible by osmotic stress and abscisic acid (ABA) [11,19,20]. During inflorescence stem development, ATHB12 represses the expression of *gibberellin 20 oxidase 1* (*GA20ox1*), thus inhibiting stem elongation [21]. ATHB12 also acts as a regulator of leaf development, promoting cell expansion in leaves as well as elevated ploidy levels [7]. ATHB7 and ATHB12 have high identity of amino acids to each other, displaying overlapping functions including water stress responses [22,23]. How ATHB12 is controlled during leaf development is not known. 

To explore the regulation of *ATHB12* expression during leaf development, we use the yeast one-hybrid method to identify upstream regulators. One of candidate genes identified as binding to the promoter of *ATHB12* in the assay, is TCP13, a member of TEOSINTE BRANCHED1/CYCLOIDEA/PCF (TCP) transcription factors, the role of which in coordinating cell division and cell differentiation during leaf development has been well established [24,25,26]. Class I TCPs stimulate cell proliferation by promoting the expression of genes involved in cell division, while class II TCPs affect leaf differentiation rather than the mitotic cycle. Increased expression of *TCP4*, which belongs to the class II, results in reduced leaf size, indicating that CINCINNATA-like TCPs (CIN-TCPs) are negative regulators of leaf growth [27,28]. In addition, TCP4 acts directly on HAT2, an HD-Zip II transcription factor, to control leaf maturation [29]. TCP13 belongs to the class II type TCPs, but its exact function in the leaf development control has not been elucidated.

Our results in this study reveal that TCP13 bound to the *ATHB12* promoter and negatively regulated *ATHB12* expression. Its overexpression resulted in a reduction of leaf cell size, suggesting that it inhibits cell expansion during leaf growth. 

## 2. Materials and Methods 

### 2.1. Plant Materials and Growth Conditions

*Arabidopsis thaliana* seeds were surface-sterilized and grown on half-strength Murashige and Skoog (MS) medium supplemented with 1% sucrose and 0.8% phytoagar. Seeds were incubated at 4 °C for 2 days and transferred to a growth chamber at 22 °C under long-day condition with a light intensity of 50 μmol m^−2^ s^−1^. 

### 2.2. Vector Construction and Plant Transformation

To generate transgenic plants overexpressing *TCP13*, full-length *TCP13* was fused with *GFP* under the cauliflower mosaic virus (CaMV) 35S promoter. An artificial microRNA (amiR) against three *TCPs* (*TCP13*, *TCP5* and *TCP17*) was made according to Efroni et al. [24]. To examine *TCP13* expression, an upstream segment (2,969-bp) of *TCP13* was amplified by PCR and inserted in front of *GUS* in pBI121. All the resulting constructs were introduced into *Agrobacterium tumefaciens* strain GV3101 and transgenic plants were obtained by the floral dip method [30].

### 2.3. Yeast One-Hybrid (Y1H) Screening and Yeast Two-Hybrid (Y2H) Assays

For yeast one-hybrid screening, a dual reporter consisting of the upstream region of *ATHB12* in pHISi-1 (*P_ATHB12_::HIS3*) and pLacZi (*P_ATHB12_::LacZ*) was constructed and integrated into *S. cerevisae* strain YM4271 (*MATa, ura3–52, his3-Δ200, ade2–101, ade5, lys2–801, leu2–3,112, trp1–901, tyr1–501, gal4Δ, gal80Δ, ade5::hisG*). A yeast strain harboring *P_ATHB12_::HIS3* was transformed with a cDNA library of 1500 *Arabidopsis* transcription factors [31] and grown on SD/-His/-Leu agar medium containing 20 mM or 60 mM 3-amino-1,2,4-triazole (3-AT). To confirm that the isolated transcription factor binds to the upstream region of *ATHB12* in yeast, a full-length cDNA of *TCP13* was cloned into pGAD424 and the resulting plasmid was introduced into the yeast strain with *P_ATHB12_::HIS3*. For Y2H assays, the Matchmaker two-hybrid system (Takara, Shiga, Japan) was used as previously reported [32].

### 2.4. Bimolecular Fluorescence Complementation (BiFC)

The coding regions of *TCP13, TCP5, TCP17* and *TCP7* were subcloned into p326-YFP^C^ vector, and those of *ATHB2, ATHB4, HAT3* and *ATHB53* were inserted into p326-YFP^N^ vector. Pairs of TCPs-YFP^C^ and ATHBs-YFP^N^ plasmids were co-introduced into *Arabidopsis* protoplasts by the PEG-method [33], and nuclei were stained with 4′,6-diamidino-2-phenylindole (DAPI). Fluorescence signals were observed after incubation for 16 h.

### 2.5. Microscopic Observation

*Arabidopsis* leaves were fixed in formaldehyde-acetic acid-alcohol (FAA) and cleared in chloral hydrate solution [7,34]. Cells of cleared tissues were observed by differential interference contrast (DIC) microscopy (Carl Zeiss, LSM700, Oberkochen, Germany). Photographs of cells at about a quarter from the bottom of the leaf, and halfway between the leaf margin and the mid-vein [35] were taken and used in measuring the cell areas. Leaves and cell sizes were measured with Image J software (http://rsb.info.nih.gov/ij). 

### 2.6. Real-Time Quantitative PCR

RNA was extracted from leaves or whole seedlings using RNeasy plant mini kit (Qiagen, Germantown, MD, USA). cDNA was synthesized using the isolated RNA by M-MLV reverse transcriptase (Promega, Madison, WI, USA). Real-time quantitative PCR was performed with qPCRBIO SyGreen Blue Mix (PCR Biosystems, London, UK) using a LightCycler 96 (Roche, Mannheim, Germany). mRNA levels were normalized with *UBQ5* and *PP2A*. Some of the gene-specific primers used in real-time PCR were reported previously [7] and others are listed in Table S1.

### 2.7. GUS Assay

GUS staining was performed as described previously [7]. Seedlings, floral organs and mature leaves were incubated in GUS staining solution containing 0.5 mg/mL 5-bromo-4-chloro-3-indolyl-β-D-glucuronic acid (X-Gluc) and cleared through acetone and ethanol series. To examine the effect of TCP13 on *ATHB12* expression, plasmids containing *GFP, TCP13-GFP* or *ATHB2-GFP* under the control of the CaMV 35S promoter were introduced into protoplasts from leaves of *P_ATHB12_::GUS* plants, and proteins from the transfected protoplasts were prepared in GUS extraction buffer (50 mM sodium phosphate buffer, pH 7.0, 10 mM EDTA, pH 8.0, 0.1% SDS and 0.1% Triton X-100). GUS activity was measured using 1 mM 4-methylumbellyferyl-β-D-glucuronide (4-MUG) in GUS extraction buffer. GUS activity was normalized with protein concentrations measured by the Bradford assay. 

### 2.8. Chromatin Immunoprecipitation (ChIP)-qPCR

Ten-day-old seedlings of *P_35S_::GFP* and *P_35S_::TCP13-GFP* were used for ChIP-qPCR. After crosslinking in 1% formaldehyde, sheared chromatin complexes were obtained by sonication (Bioruptor TOS-UCD-300; Cosmo Bio, Tokyo, Japan). Protein-DNA complexes were immunoprecipitated with GFP antibody (Abcam, Cambridge, MA, USA), and real-time PCR was performed using the primers listed in Table S1.

### 2.9. Luciferase Assay

The *P_35S_::GFP* or *P_35S_::TCP13-GFP* or *P_35S_::ATHB2-GFP* were used as effector plasmids, *P_ATHB12_::LUC* or mutated *P_ATHB12_::LUC* as reporter genes, and *Renilla* luciferase (*P_35S_::RLUC*) as internal control. *Arabidopsis* protoplasts were isolated and transfected by the polyethylene glycol (PEG)-method, as previously described [33]. Firefly luciferase (LUC) activity was normalized with *Renilla* luciferase (RLUC) activity.

## 3. Results

### 3.1. TCP13 Is an Upstream Regulator of ATHB12

**Figure 1:**
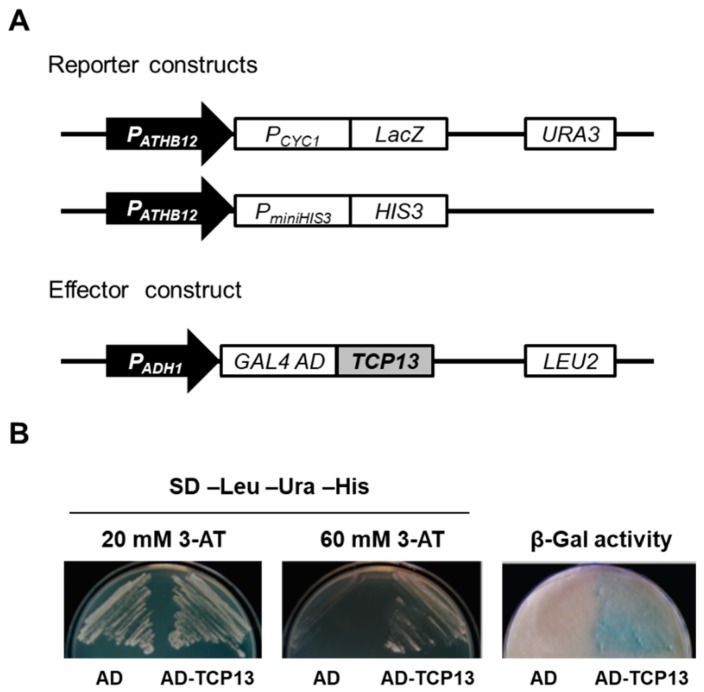
Isolation of TCP13 as an upstream regulator of *ATHB12* by yeast one-hybrid screening. (**A**) Schematic diagrams of reporter constructs. A 1.2 kb-upstream region of *ATHB12* was inserted in front of *HIS3* (pHISi-1; *P_ATHB12_::HIS3*) or *LacZ* (pLacZi; *P_ATHB12_::LacZ*), and they were used as reporter genes. *TCP13* cDNA was used as the effector construct. (**B**) TCP13 binding to the *ATHB12* promoter. Yeast cells harboring the indicated constructs were grown in the presence of 20 mM (left) or 60 mM (middle) 3-AT. Filter-lift assays (right) of the yeast cells was performed to determine β-galactosidase activities. AD; GAL4 activation domain, AD-TCP13; a fusion of GAL4 AD to TCP13.

Leaf growth involves complex regulation by many transcription factors at various stages of development. We showed previously that *ATHB12*-overexpressing plants had enlarged leaves with enlarged and endoreduplicated cells [7]. We therefore wished to know how *ATHB12* expression is regulated. To identify a direct upstream regulator(s) of *ATHB12* during leaf development, we used a yeast one-hybrid (Y1H) screen involving a cDNA library of 1500 *Arabidopsis* transcription factors [31]. A 1.2-kb upstream segment of *ATHB12* was cloned into pHISi-1 and pLacZi vectors, and the resulting reporter plasmids were used to screen the cDNA library. Twelve genes were isolated from the screen and regarded as putative upstream regulators of *ATHB12* (Table S2). We noted that three of them, TCP13, HB51 and NGA1, are known to be active in leaf development [24,36,37], and TCP13 was chosen first for this study because it is a member of the transcription factor family whose critical roles in leaf formation is well publicized [24,38]. First of all, the specific binding of TCP13 to *ATHB12* promoter was confirmed by repeating the yeast one-hybrid assay with two different stringency levels of the 3-amino-1,2,4-triazole (3-AT) selection (Figure 1). TCP13 was fused to GAL4 AD (AD-TCP13), and GAL4 AD alone (AD) was used as negative control. In the presence of 20 mM 3-AT, autoactivation of the reporter construct alone was observed but this background was effectively eliminated with 60 mM 3-AT, showing the *ATHB12* expression only in the presence of TCP13 in the assay (Figure 1B). This result strongly suggested that TCP13 is an upstream transcription factor controlling the *ATHB12* expression probably via direct binding to its promoter region.

### 3.2. Alterations of TCP13 Level Affect Leaf Morphology

**Figure 2:**
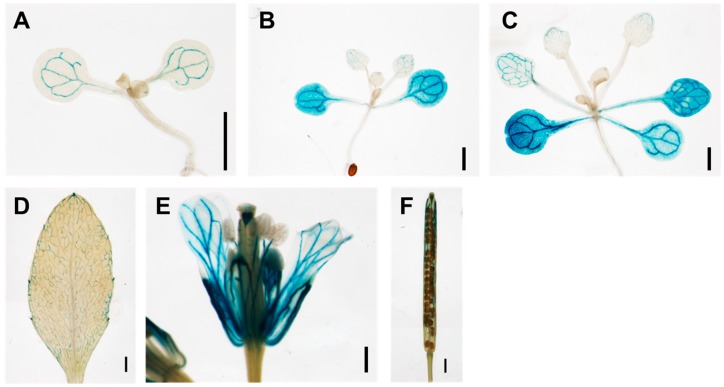
Expression of *TCP13* in planta. Seven-day-old (**A**), 10-day-old (**B**) and 16-day-old (**C**) seedlings, mature rosette leaves (**D**), flowers (**E**), and silique (**F**) of transgenic plants with a 2969-bp upstream region of *TCP13* were stained with X-gluc. Scale bars = 1 mm.

To understand the role of TCP13 in leaf development, we first analyzed its spatial and temporal expression using transgenic plants expressing a *P_TCP13_::GUS* construct composed of a 2969-bp upstream region of *TCP13* and the *GUS* gene. *TCP13* expression was detected in cotyledons, leaves, petals and siliques (Figure 2). Intriguingly, *TCP13* expression was high in cotyledons, but almost undetectable in actively dividing L3 and L4 leaves and the early expanding L1 and L2 leaves of 10-day-old plants (Figure 2B). This was the exact opposite of *ATHB12* expression during the expansion phase of leaf development; thus, in 11-day-old plants *ATHB12* was not expressed in cotyledons but highly expressed in true L1 and L2 leaves in the early expansion stage (Figure 4B in Hur et al. [7]). *TCP13* expression was also observed in the older leaves such as L1 and L2 of 16-day-old plants (Figure 2C). These observations suggested that TCP13 might inhibit the expression of *ATHB12* in leaf development.

**Figure 3:**
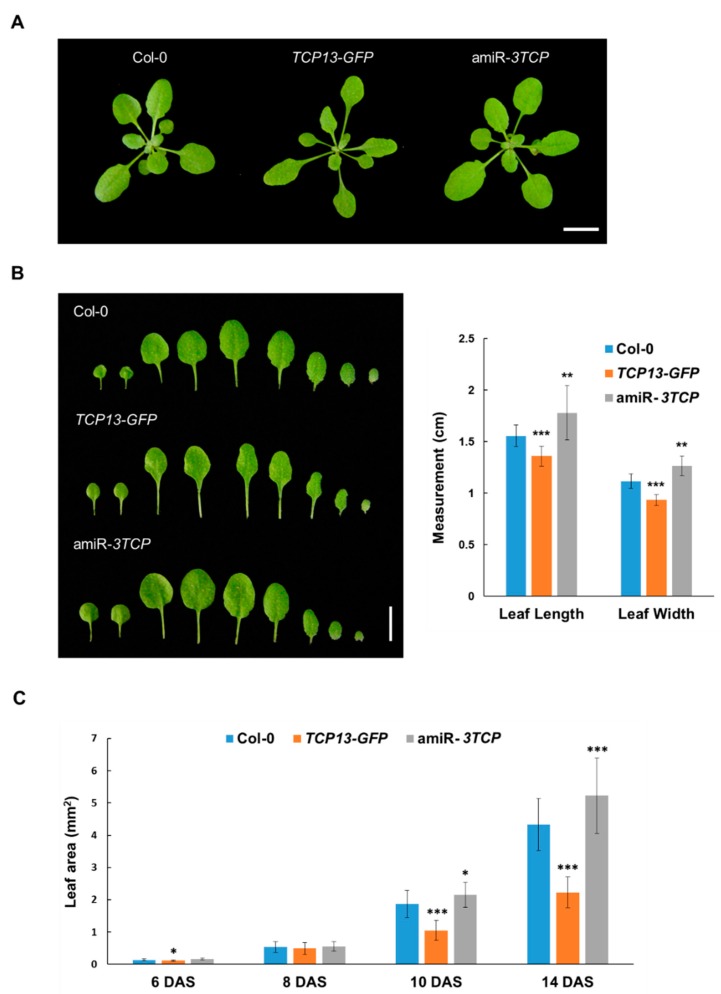
Phenotypes of *TCP13-GFP* (*P_35S_::TCP13-GFP*) and amiR-*3TCP* (*P_35S_::*amiR-*3TCP*) seedlings of *Arabidopsis thaliana*. (**A**) Growth phenotypes of four-week-old wild-type, *TCP13-GFP* and amiR-*3TCP* plants. (**B**) Leaves of four-week-old wild-type, *TCP13-GFP* and amiR-*3TCP* plants. Scale bars = 1 cm. Right panel indicates lengths and widths of fifth leaves (L5) of wild-type, *TCP13-GFP* and amiR-*3TCP* plants. Data shown are means ± SD (*n* > 8). (**C**) Area of the L1 of 6-, 8-, 10- and 14-day-old wild-type, *TCP13-GFP* and amiR-*3TCP* seedlings. DAS, days after sowing. Data shown are means ± SD (*n* > 6). Significant differences as evaluated by one-way ANOVA: ***, *p* < 0.005, **, *p* < 0.01 and *, *p* < 0.05.

Subsequently, we examined the effects of TCP13 on leaf development using transgenic plants either overexpressing *TCP13* under the cauliflower mosaic virus (CaMV) 35S promoter (*TCP13-GFP*) or expressing a construct down-regulating *TCP13* and its homologs. For the latter purpose, since a T-DNA insertional mutant of *TCP13* did not have a distinctive phenotype and in addition, it has been reported that the sequences of TCP5 and TCP17 are highly homologous to that of TCP13 [39], we made use of an artificial microRNA against *TCP5*, *TCP13*, and *TCP17* under the control of CaMV 35S promoter (amiR-*3TCP*; Efroni et al. [24]). We observed that the *TCP13* overexpressor had reduced rosette leaf laminas, whereas amiR-*3TCP* plants had enlarged rosette leaves (Figure 3A,B). Both the lengths and widths of fifth leaves of the *TCP13* overexpressor were significantly reduced whereas the leaves of amiR-*3TCP* plants were longer and wider than normal. Evidently, TCP13 and its paralogs are involved in controlling leaf size.

To examine further the role of TCP13, we measured the sizes of first leaves (L1) at different phases of leaf growth: leaf primordia (6 days after sowing; 6 DAS), cell proliferation (8 DAS) and cell expansion (10 and 14 DAS) phases [6]. The *TCP13* overexpressor had the most clear-cut effect in the cell expansion phases (10 and 14 DAS); the leaf areas of *TCP13* overexpressor were about half of those of the wild type (0.56-fold at 10 DAS and 0.51-fold at 14 DAS) whereas the amiR-*3TCP* seedlings had leaves that were about 1.2-fold larger than those of the wild type at the same stages (Figure 3C). These results indicated that TCP13 and its paralogs affected leaf growth, possibly at the cell expansion phase of leaf development. 

### 3.3. TCP13 Controls Leaf Growth Mainly by Repressing Cell Expansion

**Figure 4:**
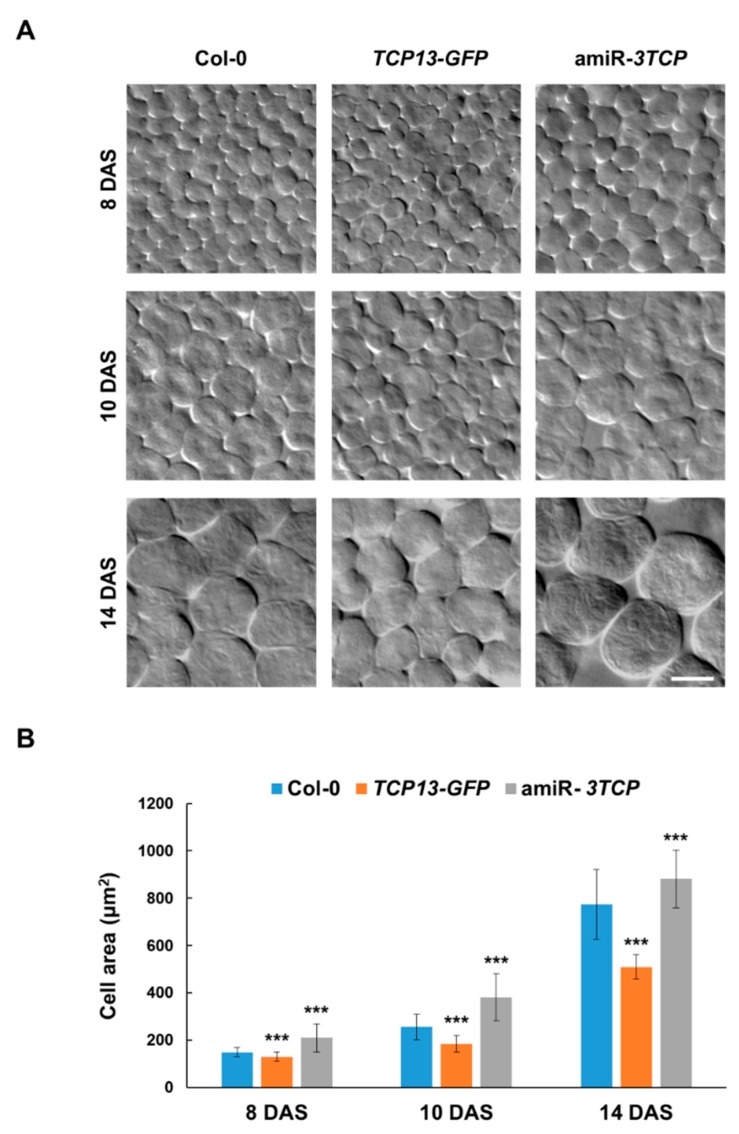
Effect of TCP13 on cell morphology of *A. thaliana*. (**A**) Microscopic observation of the palisade cells of the L1 of 8-, 10-, and 14-day-old wild-type, *TCP13-GFP* and amiR-*3TCP* seedlings. DAS, days after sowing. Scale bar = 20 μm. (**B**) Areas of the palisade cells of the L1 of 8-, 10-, and 14-day-old wild-type, *TCP13-GFP* and amiR-*3TCP* seedlings. Areas of more than 180 cells from eight leaves were measured. Data shown are means ± SD (*n* > 180). Significant differences as evaluated by one-way ANOVA: ***, *p* < 0.005, **, *p* < 0.01 and *, *p* < 0.05.

Leaf growth occurs through cell proliferation followed by cell expansion [6]. To determine whether the effect of TCP13 on leaf size is due to cell division or cell expansion, we measured the areas of the palisade cells of the *TCP13* overexpressors and amiR-*3TCP* plants at different phases of leaf development using differential interference contrast (DIC) microscopy. The palisade cells of the *TCP13* overexpressor were slightly smaller than those of the wild-type (0.86-fold) at 8 DAS, while those of the amiR-*3TCP* plants were slightly larger (Figure 4A,B). At 10 and 14 DAS, the *TCP13* overexpressor had 0.72- and 0.66-fold reduced cells, respectively. Conversely, the amiR-*3TCP* seedlings had 1.5-fold enlarged palisade cells at 10 DAS, and 1.1-fold enlarged palisade cells at 14 DAS. The areas of the palisade cells of the transgenic plants were significantly different from wild type at all the stages of leaf development examined including 8 DAS. The effect of TCP13 on leaf cell number was examined in 14-day-old L1 of *TCP13*-overexpressing plants (Figure S1). Unexpectedly, cell number was reduced at this stage, implying a complex role of TCP13 in leaf development. Taken together, our data suggested that TCP13 and its paralogs reduce leaf size primarily at the cell expansion phase of leaf development.

### 3.4. TCP13 Negatively Controls ATHB12 Expression

**Figure 5:**
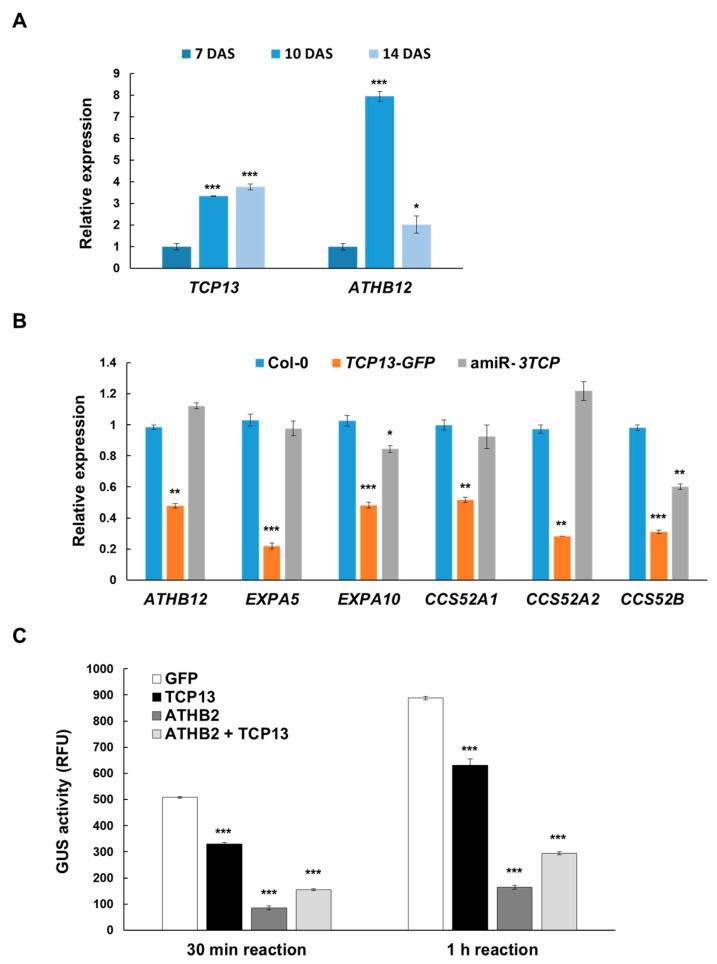
Effects of TCP13 on *ATHB12* expression. (**A**) Relative expression of *TCP13* and *ATHB12* in the L1 and L2 of 7-, 10-, and 14-day-old wild-type seedlings. Results are representative of more than three independent experiments. Data shown are means ± SD (*n* = 2). (**B**) Relative expression of *ATHB12, EXPA5, EXPA10,* and *CCS52s* in 14-day-old wild-type, *TCP13-GFP* and amiR-*3TCP* plants examined by real-time quantitative PCR. Results are representative of more than three independent experiments. Data shown are means ± SD (*n* = 2). (**C**) GUS activities in protoplasts isolated from transgenic plants with P*_ATHB12_::GUS* was measured after transfection with *P_35S_::GFP* or *P_35S_::TCP13-GFP* or *P_35S_::ATHB2-GFP* constructs. Data shown are means ± SD (*n* = 3). RFU, Relative fluorescence units. Significant differences as evaluated by one-way ANOVA: ***, *p* < 0.005, **, *p* < 0.01 and *, *p* < 0.05.

Phenotypes of *TCP13-GFP* and amiR-*3TCP* plants indicated that TCP13 inhibits the leaf growth mainly at the cell expansion phase. Furthermore, it was previously shown that ATHB12 promotes the cell expansion of leaves through endoreduplication [7]. We hypothesized that TCP13, as a transcription factor, binds to the upstream region of *ATHB12* and regulates its expression. To test this idea, we measured the expression of *TCP13* and *ATHB12* at different phases of leaf development. The expression of *TCP13* was significantly increased at 10 DAS and the trend continued at 14 DAS as well, although with a much-reduced rate. On the other hand, the *ATHB12* expression showed its peak at 10 DAS then fell sharply at 14 DAS to about ¼ of the level observed at 10 DAS (Figure 5A). This seemed to be in agreement with the idea of the *ATHB12* expression being negatively regulated by TCP13.

We wanted to verify the negative correlation of expression between *ATHB12* and *TCP13*, so that the expression of *ATHB12* was also examined in the aforementioned transgenic plants by real-time PCR. Consistent with what was observed in the wild-type plants, it was found to be reduced in the leaves of transgenic plants overexpressing *TCP13* while slightly increased expression level was detected in the leaves of amiR-*3TCP* plants, supporting our proposed role of TCP13 and its paralogs in suppressing the *ATHB12* expression (Figure 5B). In the meantime, there was no significant difference between the levels of *ATHB12* expression in the two types of transgenic plants when RNAs from whole seedlings were used (Figure S2), implying that TCP13 appears to affect *ATHB12* expression only in leaves. Furthermore, *EXPA5* and *EXPA10*, expansion genes whose expression is induced by ATHB12 [7], were downregulated in *TCP13*-overexpressing plants (Figure 5B). In addition, the expression of *CCS52s*, marker genes for endoreduplication [40], were significantly decreased in *TCP13*-overexpressing plants and increased in amiR-*3TCP* plants. These patterns of expression were confirmed in *TCP13-GR* seedlings induced with dexamethasone (DEX) in the presence of cycloheximide for 2 h (Figure S3).

**Figure 6:**
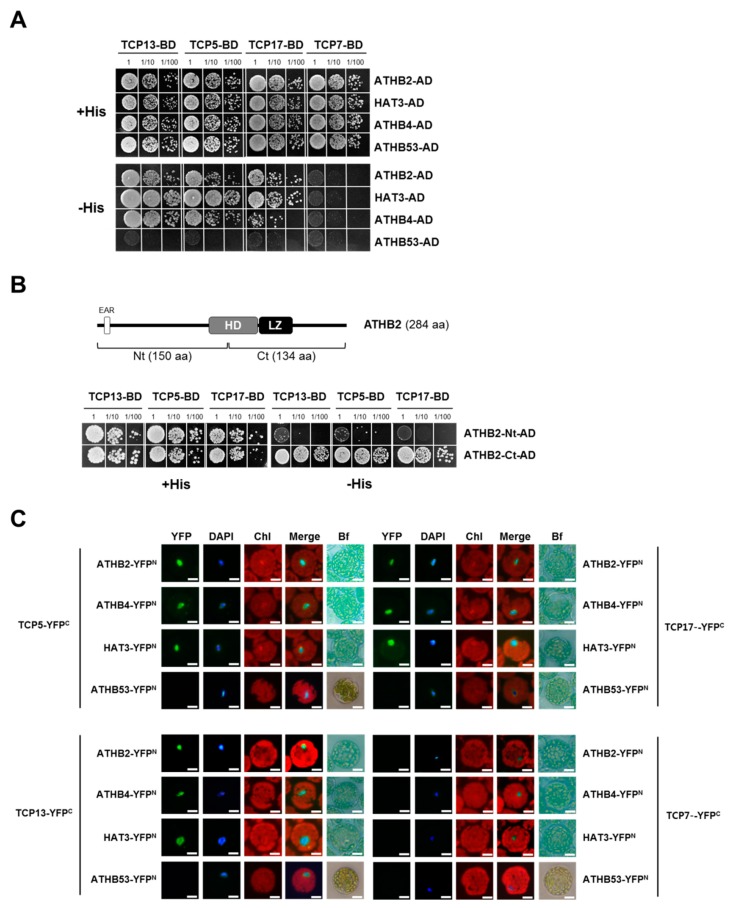
Interaction of TCP13 with HD-Zip class II proteins. (**A**) TCP13 and TCP13 homologous CIN-TCPs interact with HD- Zip class II protein in yeast two-hybrid assay. Dilutions of yeast transformed with TCP-BD and ATHB-AD were spotted in medium with the presence or absence of histidine (His). TCP7, a member of TCP class I, fused to BD and ATHB53, a HD- Zip class I protein, fused to AD were used as negative controls. BD, binding domain; AD, activation domain. (**B**) TCP13 interacts with ATHB2 C-terminal. Top panel shows schematic representation of domain structure of ATHB2. HD, homeodomain; LZ, leucine-zipper. TCP13, TCP5, and TCP17-BD interact with ATHB2-Ct-AD, but not with ATHB2-Nt-AD in yeast two-hybrid assay. (**C**) Bimolecular fluorescence complementation (BiFC) analysis of interaction between CIN-TCPs and HD- Zip class II proteins. *Arabidopsis* protoplasts were co-transfected with several combinations of the constructs of *P_35S_::ATHB-YFP^N^* and *P_35S_::TCP-YFP^C^*. DAPI stained the nuclei. Chl, chlorophyll; Bf, bright field. BiFC experiments were replicated three times with similar results. Scale bar = 10 μm.

In order to clarify whether the change of the expression of *ATHB12* were due to TCP13, we transfected protoplasts from leaves harboring *P_ATHB12_::GUS* with a plasmid containing *GFP* alone or *TCP13-GFP* under the control of CaMV 35S promoter. GUS activities measured 0.5 h or 1 h after transfection indicated that *TCP13* overexpression indeed reduced *ATHB12* expression in the protoplasts (Figure 5C). We also examined the effect of ATHB2 on the expression of *ATHB12* because TCP13 was found to interact with ATHB2 (see Figure 6) and ATHB2 contains an EAR motif, a well-known transcriptional repression motif in plants [41]. Transfection of protoplasts of *P_ATHB12_::GUS* with a *P_35S_::ATHB2-GFP* construct reduced GUS activity more than transfection of a control *P_35S_::GFP* construct. Then for examining a possible synergistic effect of TCP13 and ATHB2 on the reduction of *ATHB12* expression, protoplasts of *P_ATHB12_::GUS* were transfected with both *P_35S_::TCP13-GFP* and *P_35S_::ATHB2-GFP* constructs. However, transfection of both TCP13 and ATHB2 did not result in a synergistic effect on the repression but a slight derepression was observed as compared with that of the ATHB2 alone. Validating these data, when these constructs were used to transfect protoplasts isolated from wild-type *Arabidopsis* leaves together with the plasmid DNA having luciferase reporter construct under the *ATHB12* promoter (*P_ATHB12_::LUC*), exactly the same results were also obtained (Figure S4). ATHB2 and TCP13 interact with each other, resulting in slight de-repression or more repression of *ATHB12*, depending on which protein binds to *ATHB12* promoter first, but at this point, the exact mechanism on how TCP13 and ATHB2 affect *ATHB12* expression requires further research.

To determine if ATHB2 can bind directly to the *ATHB12* promoter region, yeast one-hybrid analysis was conducted after fusing full-length ATHB2 coding sequence to the GAL4 activation domain (AD-ATHB2). A specific binding of ATHB2 to the promoter of *ATHB12* under high stringency selection condition (60 mM 3-AT) was observed, confirming ATHB2 as another transcription factor participating in the regulation of *ATHB12* expression possibly through direct binding to the promoter (Figure S5). All these findings supported the view that TCP13 and its paralogs as well as ATHB2 inhibit *ATHB12* expression. 

### 3.5. TCP13 Interacts with ATHB2

The *Arabidopsis* interactome map provides a comprehensive network of protein interactions in *Arabidopsis* [42]. The interactome data suggest that TCP13 interacts with ATHB2, a homeodomain-leucine zipper protein (HD-Zip) containing an EAR motif [41]. Thus, possibility of the interaction was tested by yeast two-hybrid assays and the result demonstrated that ATHB2, a HD-Zip class II protein, specifically interacts with TCP13 as well as two other CIN-like TCPs (TCP5 and TCP17) reflecting highly conserved amino acid sequence identities among these three CIN-like TCPs, as ATHB2 did not show any interaction with TCP7, a member of a different class of TCPs, in the same assay (Figure 6A). The interactions were revealed to be mediated with the C-terminal region of ATHB2 which contains a leucine zipper (Figure 6B). In addition to ATHB2, TCP13 was found to interact with other members of HD-Zip class II proteins including ATHB4 and HAT3 (Figure 6A), which were further confirmed in vivo by bimolecular fluorescence complementation (BiFC) analysis (Figure 6C). These results indicate that a close functional tie might exist between CIN-type TCPs and the class II HD-Zip proteins established at structural level. Given that ATHB2, ATHB4 and HAT3 contain an EAR motif, a plant-specific repression domain, functional significance of this interaction between TCP13 and ATHB2 in the negative regulation of *ATHB12* expression warrants more in-depth examination.

### 3.6. TCP13 Binds to the Promoter of ATHB12 in Vivo

**Figure 7:**
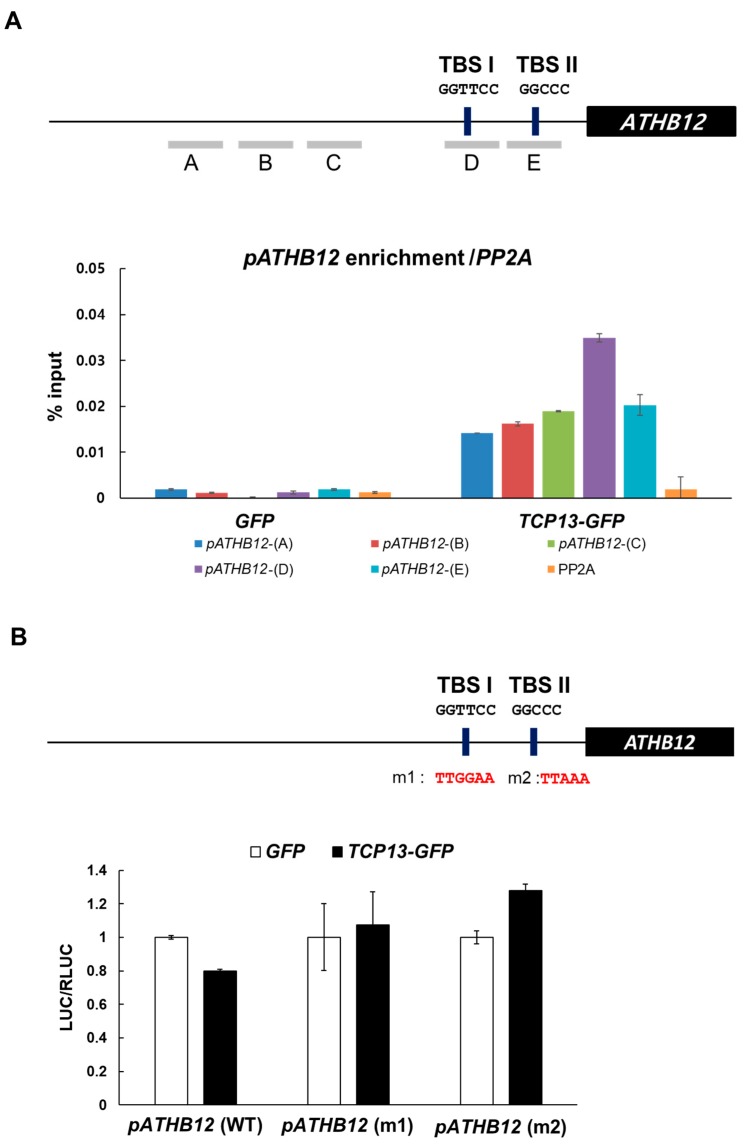
(**A**) Binding of TCP13 to the upstream region of *ATHB12*. Association of TCP13 with the upstream region of *ATHB12* was confirmed by chromatin immunoprecipitation (ChIP) assays. Schematic diagram indicates TCP binding sites (TBS I-II) and ChIP amplicons (A-E) in the upstream region of *ATHB12*. The enrichment of each DNA fragment was normalized by the level of input DNA and the ChIP enrichment value of the control *PP2A* promoter. Data shown are means ± SD (*n* = 2). (**B**) Luciferase assays using wild-type or mutated *ATHB12* promoter. The diagram shows the sequences of wild-type (WT) or mutated (m1 and m2) versions of *ATHB12* promoter. *Arabidopsis* protoplasts were transiently transfected with different reporter genes, *P_ATHB12 (WT)_::LUC* (*pATHB12* (WT) or *P_ATHB12 (m1)_::LUC* (*pATHB12* (m1)) or *P_ATHB12 (m2)_::LUC* (*pATHB12* (m2)) and, together with *P_35S_::RLUC* as internal control and effectors such as *P_35S_::GFP* or *P_35S_::TCP13-GFP* constructs. Firefly luciferase (LUC) activity was normalized with *Renilla* luciferase (RLUC) activity. Data shown are means ± SD (*n* = 2).

Since our yeast one-hybrid analysis results suggested that TCP13 regulates the expression of *ATHB12* via direct binding to the promoter region, we attempted to map the binding site on the *ATHB12* promoter. Class II TCP proteins are predicted to bind to the consensus binding site (G(T/C)GGNCCC) [25,43]. Sequence analysis revealed that the upstream region of *ATHB12* contains two potential TCP binding sites (TBSs) at -254 bp (TBS I; GGTTCC) and -158 bp (TBS II; GGCCC), respectively, from the translational start site (Figure 7A). Chromatin immunoprecipitation (ChIP) assays were employed to examine the binding of TCP13 to the upstream region of *ATHB12* including the putative TCP binding sites. ChIP assays using *TCP13-GFP* plants strongly enriched a fragment of the *ATHB12* upstream region that contained TBS I (region D in Figure 7A) whereas ChIP using transgenic plants expressing only *GFP* did not show enrichment of any *ATHB12* upstream region as negative control. These indicated that TCP13 binds to the upstream region of *ATHB12* in planta. To further verify that the TBS I and TBS II motifs on *ATHB12* promoter are the core elements to which TCP13 binds and regulates the expression of *ATHB12,* we mutagenized the TBS I and TBS II sequences (Figure 7B) and monitored the effect in protoplasts transiently expressing the luciferase reporter gene under the wild-type *ATHB12* promoter or the ones carrying these mutations. The expression of luciferase driven by wild-type *ATHB12* promoter was substantially repressed with co-transfection of *P_35S_::TCP13-GFP* as in the GUS assay shown in Figure 5C (Figure 7B). However, the luciferase activity measured from the cells transfected with either of the two mutant promoters (m1 or m2) exhibited clear de-repression under the same setting, demonstrating that the TBS I and TBS II sequence motifs are indeed the critical elements to which TCP13 convey its regulatory effect, likely through direct binding. We conclude that TCP13 regulates *ATHB12* expression through direct binding to the *ATHB12* upstream region containing TBS I and TBS II.

## 4. Discussion

Leaf development requires a controlled cascade of activities of diverse transcription factors. Previously, ATHB12, a homeodomain transcription factor, was reported to promote leaf growth, especially in the cell expansion phase, by activating several genes related to cell expansion [7]. However, how ATHB12 itself was controlled during leaf development remained unknown. In this report, we isolated and characterized an upstream regulator of *ATHB12*. 

TCP13, a class II TCP transcription factor, was isolated as an upstream regulator of *ATHB12* (Figure 1). It seems to affect the specific phase(s) of leaf growth, which is reasonable considering the role of ATHB12 in leaf growth [7]. Expression of *TCP13* was mainly observed in cotyledons, and was not detected in the first to fourth leaves of 11-day-old seedlings (Figure 2B). This pattern is exactly the opposite of *ATHB12* expression during the expansion phase of leaf development [7]. TCP13 affected the areas of leaves at 8 DAS to 14 DAS—specifically cell areas of leaves at the corresponding stages (Figure 3C and Figure 4). Class II TCP transcription factors are key regulators of leaf growth [24,29,44,45,46,47]. Timing of *TCP13* expression and the phenotypes of *TCP13* transgenic plants indicate that TCP13, one of the class II TCP family, controls a specific period(s) of leaf development including the cell expansion stage. However, given the significant differences in cell area of 8 DAS transgenic plants (Figure 4B) and in cell numbers of 14-DAS plants (Figure S1), we cannot exclude a possibility that TCP13 and its paralogs may also somehow affect cell division.

The expression of *TCP13* was examined by GUS assays of transgenic plant expressing a *P_TCP13_::GUS* reporter (Figure 2). In addition, the expression of TCP13 and ATHB12 was further studied by real-time PCRs and transactivation assays using *P_ATHB12_::GUS* (Figure 5), which suggests that TCP13 regulates *ATHB12* expression negatively. Many TCPs control gene transcription negatively in various periods of plant development. TCPs such as TCP2 and TCP3, which are regulated by miR319, bind to the promoter regions of class-I *KNOX* genes and repress their transcription [46]. TCP target genes have been shown to be repressed during ovule development by epigenetic regulation involving the SPL-TPL-HDA19 complex [48]. Furthermore, TCP5-like proteins including TCP13, control expansion of the cells of petals since overexpression of *TCP5* results in petals of reduced area [49]. TCP13, and possibly other CIN-TCPs, regulate the cell area of leaves negatively, but how they repress their target genes, which are involved in cell expansion, needs further examination. 

The expressions of *ATHB12* and its key downstream target genes were all shown to be significantly down regulated in *TCP13-GFP* expressing transgenic plants (Figure 5B). On the contrary, some of these downstream target genes (notably *CCS52B*) did not fully recover their expression levels in the in amiR-*3TCP* transgenic plants. A possible explanation for this discrepancy could be that many of these are indirect targets of ATHB12, and perhaps other additional factors as well as ATHB12 could be required for their full activation. Or the restored *ATHB12* expression in the amiR-*3TCP* plants was not completely linked to the full activation of the ATHB12 protein for some reasons. At this point, the exact explanation remains unanswered.

Interaction between TCP13 and ATHB2 was confirmed by yeast two-hybrid assays and BiFC analysis (Figure 6). In addition, TCP5 and TCP17, other members of CIN-like TCPs sharing high sequence homology with TCP13, also interact with ATHB2 in the same assays. Besides, other HD-Zip class II proteins including ATHB4 and HAT3 also showed interaction with TCP13/5/17. Therefore, it is probable that the rest of CIN-like TCPs such as TCP3 and TCP4 could also interact with ATHB2, ATHB4 and HAT3 to control the expression of *ATHB12* or other genes, considering the highly conserved structural similarity in helices and the loop domain in the CIN-like TCPs [25]. TCP4, as an example, affects leaf morphogenesis as well as other developmental phases of plant [27], so that TCP4 may associate with ATHB2 and other HD-Zip class II proteins for functional overlapping and fine-tuning in a similar fashion to other related TCPs including TCP13. A continuous and extensive effort to unveil detailed mechanisms underlying the interaction between TCPs and ATHBs could provide significant understanding on leaf development. 

We isolated several transcription factors in addition to TCP13 from the yeast one-hybrid screen for proteins that bind to the 1.2-kb upstream region of *ATHB12* (Table S2, Figure 1). Interestingly, some of them also affected the growth of leaves and flowers. NGA1 is an AtNGA family member that acts as negative regulator of the cell proliferation in lateral organs [36]. Overexpression of *NGA* resulted in reduced leaf cell numbers. In addition, *HB51*, also called *LMI1*, is expressed in expanding leaves and affects leaf morphology [37]. It is quite possible that several of the transcription factors isolated from the yeast one-hybrid screening affect *ATHB12* expression/repression, thus influencing particular phases of leaf development. Further research on them in relevance of ATHB12 should clarify their roles in leaf development.

## Supplementary Materials

The following are available online at https://www.mdpi.com/2073-4425/10/9/644/s1, Table S1: Primers used in this study, Table S2: List of putative upstream regulators of *ATHB12* isolated by yeast one-hybrid screening, Figure S1: Effect of TCP13 on leaf cell number in *Arabidopsis*, Figure S2: *ATHB12* expression in whole seedlings of wild-type, *TCP13-GFP* and amiR-*3TCP* plants, Figure S3: Expression of downstream genes of ATHB12 after TCP13 induction Figure S4: Effect of TCP13 and ATHB2 on the expression of *ATHB12* examined by luciferase assay. Figure S5: Binding of ATHB2 to the *ATHB12* promoter examined by yeast one-hybrid assay.
