# Supplementary material for: Identification of TCP13 as an Upstream Regulator of ATHB12 during Leaf Development
Source: Genes (Basel). 2019 Aug 26;10(9):644. doi: 10.3390/genes10090644 (PMC6770448; doi:10.3390/genes10090644)
Supplement: Supplementary file 1 [file genes-10-00644-s001.pdf]

Supplementary Materials:

Table S1. Primers used in this study.

Gene Cloning

Name	Forward	Reverse
P$_{TCP13}$-GUS	GGG AAG CTT AAC CTA CGA AAT TTT CTT AAC GTA AAT T	CCC TCT AGA AAT GCT ATT CTC GAG ATC TTT ATA TCT A
pATHB12::HIS3	GGG GAA TTC TTT ATT TTT CTT GGG CAA TGA AT	CTC TCT AGA GGT TTT CAC CAG ATC TTG TAA GTT
pATHB12::LacZ	GGG GAA TTC TTT ATT TTT CTT GGG CAA TGA AT	CTT CCT CGA GGG TTT TCA CCA GAT CTT GTA AGT TTC

qRT-PCR

Name	Forward	Reverse
TCP13	TTC CC AAA ACA TTT CGA CAT	TAC AAC GCT CCG AGC CTA GA
ATHB12	GGT TAG ACC AAG GGA GTG TTC TAT GT	CAA TTC TCA GAA GAT GTC AAG CAA CT
UBQ5	CGG ACC AGC AGC GAT TG	GGG TAC GGC CGT CTT CAA G
PP2A	TAA CGT GGC CAA AAT GAT GC	GTT CTC CAC AAC CGC TTG GT

ChIP-qPCR

Name	Forward	Reverse
pATHB12-(A)	ACT AAT GAA AAG TCT CGT TAA AAC	ATC AGG ATG TTT GAC GGG TGT TAA
pATHB12-(B)	ATG TGA ACG TGG AAT CTC GTT ACA	ATC ATC ATT TAA CAT GTG ACT GCC
pATHB12-(C)	ATA CGT TGT TGC ATG TTA AAA CAT	GTT CCT AAG AAA AAT GAA GCA GTT
pATHB12-(D)	AAA AGT ATG AAA ATA TTA TCA GTA	GAC CTG CAA ATA TTG TGG AAC CAC
pATHB12-(E)	TTT TTA TTC AAT GAG TTT ATT TTG	TTC TGG AAT CTC TGA GTT GCT GAT
PP2A	CTG GCG TGT GCG TTA TAT G	CAA ACA TGG ACT TCC AAG TAC C

Table S2. List of putative upstream regulators of *ATHB12* isolated by yeast one-hybrid screening.

Locus	Gene name
AT3G61120	AGAMOUS-like 13(AGL13)
AT2G46870	AP2/B3-like transcriptional factor family protein(NGA1)
AT2G45850	AT hook motif DNA-binding family protein(AT2G45850)
AT2G16400	BEL1-like homeodomain 7(BLH7)
AT3G02380	CONSTANS-like 2(COL2)
AT1G02210	NAC domain transcriptional regulator superfamily protein(AT1G02210)
AT5G18300	NAC domain containing protein 88(NAC088)
AT5G07500	Zinc finger C-x8-C-x5-C-x3-H type family protein(PEI1)
AT1G35240	auxin response factor 20(ARF20)
AT2G22760	basic helix-loop-helix (bHLH) DNA-binding superfamily protein(AT2G22760)
AT5G03790	homeobox 51(HB51)
AT3G02150	plastid transcription factor 1(PTF1)/ TEOSINTE BRANCHED1, CYCLOIDEA AND PCF TRANSCRIPTION FACTOR 13 (TCP13)

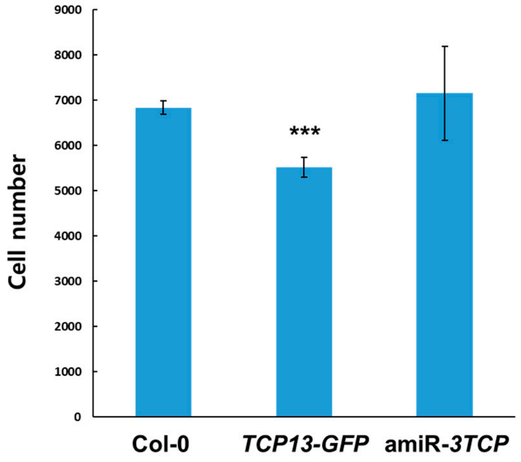

Figure S1. Effect of TCP13 on leaf cell number in *Arabidopsis.* Cell number of the L1 of 14-day-old wild-type, *TCP13-GFP* and amiR-*3TCP* seedlings. Significant differences as evaluated by one-way ANOVA: ***, $P < 0.005$, **, $P < 0.01$ and *, $P < 0.05$. Data shown are means ± SD ($n = 5$).

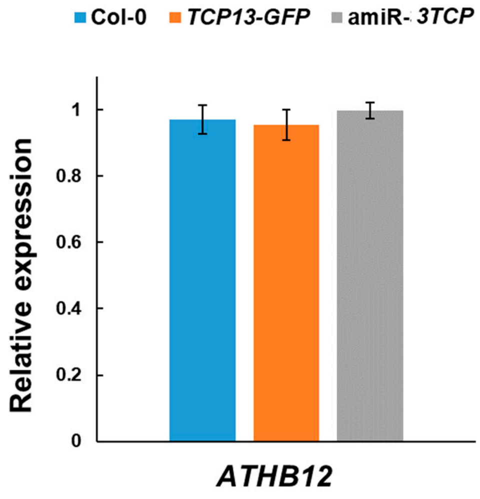

Figure S2. *ATHB12* expression in whole seedlings of wild-type, *TCP13-GFP* and amiR-3*TCP* plants. Expression of *ATHB12* in the wild-type, *TCP13-GFP* and amiR-3*TCP* seedlings was examined by real-time quantitative PCR using the whole seedlings. Results are representative of more than three independent experiments. Data shown are means ± SD (n = 2).

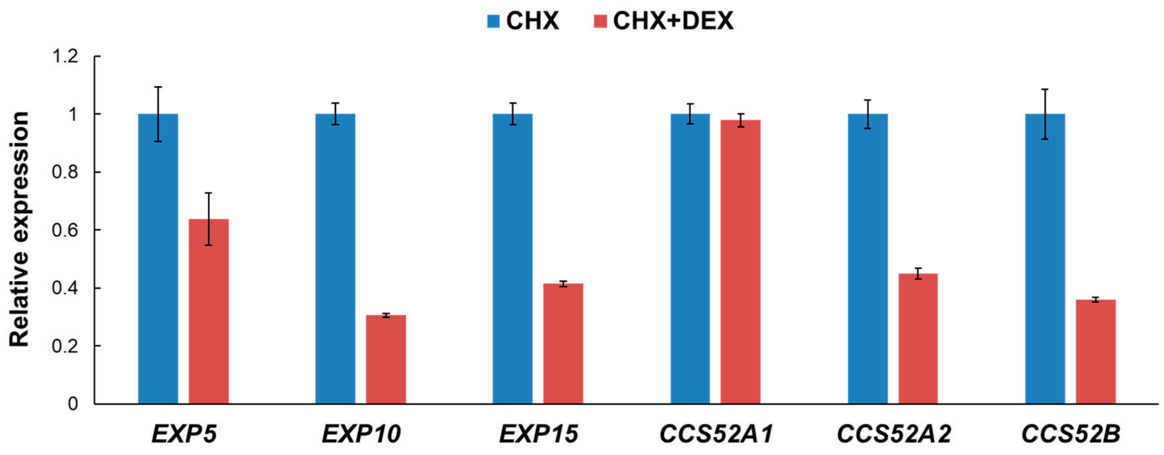

Figure S3. Expression of downstream genes of ATHB12 after TCP13 induction. Effect of TCP13 induction on the expression of *ATHB12* and *EXPANSINs* (*EXPAs*) was examined by real-time quantitative PCR. Total RNAs was isolated from *TCP13-GR* seedlings with or without 10 μM dexamethasone (DEX) in the presence of 10 μM cycloheximide for 2 h. Results are representative of more than three independent experiments. Data shown are means ± SD (n = 2).

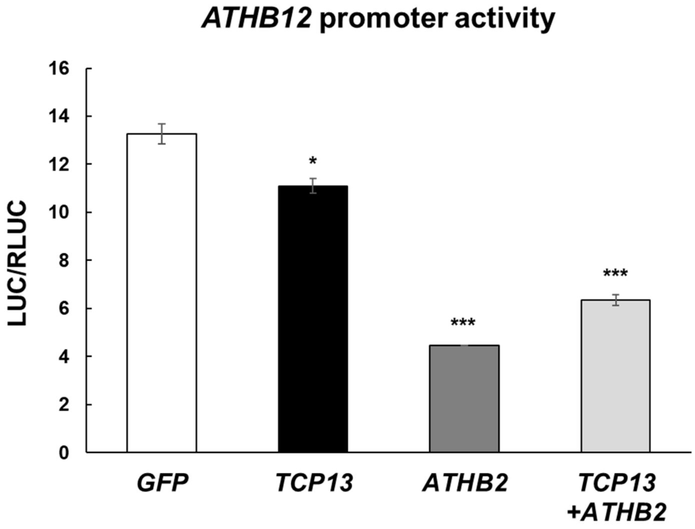

Figure S4. Effect of TCP13 and ATHB2 on the expression of *ATHB12* examined by luciferase assay. Luciferase assays using a 2.2-kb region of *ATHB12* promoter. *Arabidopsis* protoplasts were transiently transfected with reporter genes, P_{ATHB12}*::LUC* and P_{35S}*::RLUC*, together with several effectors such as P_{35S}*::GFP* or P_{35S}*::TCP13-GFP* or P_{35S}*::ATHB2-GFP* constructs. Firefly luciferase (LUC) activity was normalized with *Renilla* luciferase (RLUC) activity. Data shown are means ± SD (n = 2). Significant differences as evaluated by one-way ANOVA: ***, $P < 0.005$, **, $P < 0.01$ and *, $P < 0.05$.

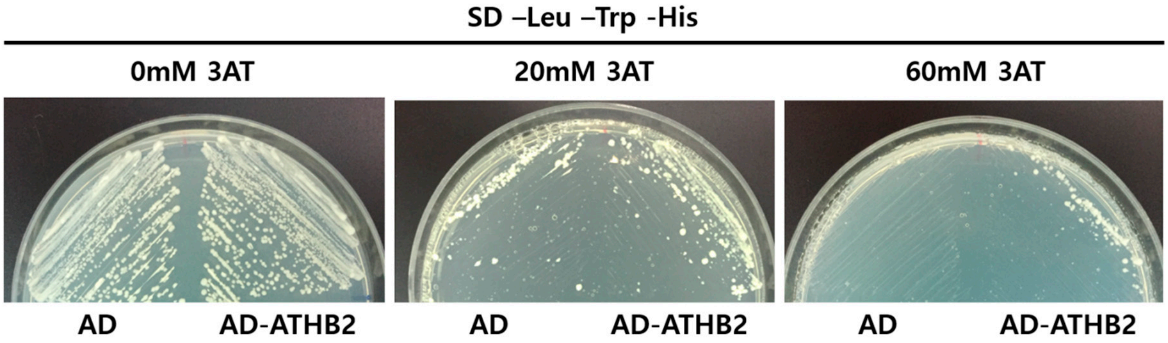

Figure S5. Binding of ATHB2 to the *ATHB12* promoter examined by yeast one-hybrid assay. Full-length cDNA of *ATHB2* was in-frame fused to GAL4 AD domain of pGAD424 and the resulting vector (AD-ATHB2) or the empty vector (AD) was introduced into *S. cerevisae* strain AH109 (*MATa, trp1-901, leu2-3, 112, ura3-52, his3-200, gal4Δ, gal80Δ, LYS2::GAL1*$_{UAS}$*-GAL1*$_{TATA}$*-HIS3, MEL1, GAL2*$_{UAS}$*-GAL2*$_{TATA}$*-ADE2, URA3::MEL1*$_{UAS}$*-MEL1*$_{TATA}$*-lacZ*) containing P_{ATHB12}*::His* construct. Transformants were grown on SD/-Leu/-His/-Trp agar medium in the presence of 20 mM (middle) or 60 mM (right) 3-AT for 2 ~ 3 days at 30℃.
